# Microarray and qPCR Analysis of Mitochondrial Metabolism Activation during Prenatal and Early Postnatal Development in Rats and Humans with Emphasis on CoQ_10_ Biosynthesis

**DOI:** 10.3390/biology10050418

**Published:** 2021-05-08

**Authors:** Jana Krizova, Martina Hulkova, Vaclav Capek, Petr Mlejnek, Jan Silhavy, Marketa Tesarova, Jiri Zeman, Hana Hansikova

**Affiliations:** 1 Laboratory for Study of Mitochondrial Disorders, Department of Pediatrics and Inherited Metabolic Disorders, First Faculty of Medicine, General University Hospital in Prague, Charles University, Ke Karlovu 2, 128 08 Prague 2, Czech Republic; jana.krizova@vfn.cz (J.K.); mar.hulkova@gmail.com (M.H.); venca@ciconia.cz (V.C.); marketa.tesarova@lf1.cuni.cz (M.T.); jzem@lf1.cuni.cz (J.Z.); 2 Department of Genetics of Model Diseases, Institute of Physiology AS CR, v.v.i., Videnska 1083, 142 20 Prague 4, Czech Republic; petr.mlejnek@fgu.cas.cz (P.M.); jan.silhavy@fgu.cas.cz (J.S.)

**Keywords:** mitochondria, coenzyme Q, ubiquinone, microarray, prenatal, human, rat, qPCR

## Abstract

**Simple Summary:**

We lack studies investigating mitochondrial metabolism in the prenatal and early postnatal period in humans, but parallel experiments conducted in a mammalian system are informative about the human condition. Our aim was to study the perinatal metabolic switch in rats—an extremely complex process, associated with tissue proliferation and differentiation together with a rapid oxidative stress response (using techniques including microarrays, qPCR, spectrophotometry and high-performance liquid chromatography). Out of 1546 mitochondrial genes, 1119 and 827 genes significantly changed expression in rat liver and skeletal muscle, respectively. The most remarkable expression shift occurred in the rat liver at least two days before birth. Coenzyme Q and mitochondrial metabolism-based evaluation in both the rat model and human tissues showed the same trend: the total CoQ content and mitochondrial metabolism significantly increases after birth, possibly regulated by COQ8A kinase. Our microarray data could serve as a suitable background for finding key factors regulating mitochondrial metabolism and preparation of the foetus for the transition to extra-uterine conditions, or as preliminary data for further studies of the complex mitochondrial metabolism regulation and diagnostics of mitochondrial disorders.

**Abstract:**

At the end of the mammalian intra-uterine foetal development, a rapid switch from glycolytic to oxidative metabolism must proceed. Using microarray techniques, qPCR, enzyme activities and coenzyme Q content measurements, we describe perinatal mitochondrial metabolism acceleration in rat liver and skeletal muscle during the perinatal period and correlate the results with those in humans. Out of 1546 mitochondrial genes, we found significant changes in expression in 1119 and 827 genes in rat liver and skeletal muscle, respectively. The most remarkable expression shift occurred in the rat liver at least two days before birth. Coenzyme Q-based evaluation in both the rat model and human tissues showed the same trend: the total CoQ content is low prenatally, significantly increasing after birth in both the liver and skeletal muscle. We propose that an important regulator of rat coenzyme Q biosynthesis might be COQ8A, an atypical kinase involved in the biosynthesis of coenzyme Q. Our microarray data, a total of 16,557 RefSeq (Entrez) genes, have been deposited in NCBI’s Gene Expression Omnibus and are freely available to the broad scientific community. Our microarray data could serve as a suitable background for finding key factors regulating mitochondrial metabolism and the preparation of the foetus for the transition to extra-uterine conditions.

## 1. Introduction

Mitochondria are key players in mammalian ATP production. Oxidative phosphorylation, the mitochondrial process which produces the majority of cellular ATP, is executed by a system of several supercomplexes (OXPHOS) attached to the mitochondrial inner membrane. Regulation of ATP production is a complex process involving mitochondrial DNA (mtDNA) replication and transcription, the expression of OXPHOS subunits, biosynthesis of electron carriers (e.g., coenzyme Q, CoQ; cytochrome c), or even mitochondrial fission–fusion machinery regulation. Moreover, the regulation of genes encoded both in nuclear DNA (nDNA) and mtDNA must be precisely orchestrated. Therefore, it has to be executed by a number of regulators.

Dynamic changes in mitochondrial metabolism have been described throughout foetal development, and especially during the rapid perinatal switch to the extra-uterine conditions, in order to meet the energy demands in various tissues [1]. Foetal metabolism has been described as based on glycolysis [2], because the partial oxygen pressure in utero is low [3]. After birth, the concentration of ATP in the rat liver increases two-fold within the first two hours [4]. This metabolic switch has also previously been reported in other tissues and organisms [5,6,7,8,9], encompassing changes in OXPHOS activities and mtDNA content as well. Additionally, various tissues show a distinct stoichiometry of the OXPHOS complexes [10,11]. On the other hand, the pool of CoQ and cytochrome c is generally in excess of the complexes [12]. In humans and other mammals, the predominant CoQ form has 10 isoprenoid residues (hence, CoQ_10_), but in rodents, the predominant form has only nine isoprenoid residues (CoQ_9_) [13]. How the synthesis of the distinct CoQ forms is regulated is not well understood, but the higher CoQ_9_/CoQ_10_ ratio is believed to be an adaptation to oxidative stress in various rodent models [14,15,16,17]. In humans, the accumulation of CoQ_10_ during the first days and weeks of life is physiological, although the initial levels at birth are lower compared to adult levels [18]. At birth, preterm newborns exhibit higher levels of oxidative stress (higher levels of hydroperoxides in plasma and higher lipid peroxidation; lower levels of vitamins A, C, E, and superoxide dismutase). CoQ_10_ seems to play role in protecting the cell membranes and lipoproteins against lipid peroxidation in the antioxidant system of the plasma membrane, together with the NADH-cytochrome b5 and NADPH-cytochrome P450 reductases, and vitamins A, C and E [14,19,20,21].

Among the risks which arise from increased perinatal oxidative stress, usually in preterm newborns, we can mention respiratory distress syndrome, bronchopulmonary dysplasia, periventricular leukomalacia, necrotizing enterocolitis, patent ductus arteriosus, and retinopathy [22,23,24]. However, term newborns are also vulnerable to oxidative damage of cell membranes leading to brain injury, and possibly later to neurodevelopmental disorders, asthma, diabetes mellitus, hypertension, coronary heart disease, and stroke [25]. In human samples, oxidative stress is assessed mainly in the umbilical cord blood, urine, or plasma [26]. Several therapeutic strategies have been suggested for newborns suffering from oxidative stress: hypothermia, free radical production inhibition, excitatory amino acid antagonists, nitric oxide inhibition, stem cell therapy, mitochondrial therapy, and hyperbaric oxygen therapy [27,28]. Direct molecular therapy targeting mitochondria was proposed in studies using metformin and mitoquinone, which prevent inflammation and hypoxic brain injury, respectively. The treatment by exogenous CoQ_10_ administration showed promising results in models of Alzheimer’s disease, hyperglycaemia, and traumatic brain injury, but not in the neonatal ischemia [27]. Hence, after the elucidation of CoQ_10_ biosynthesis regulation, a new approach to treating neonatal oxidative stress by enhancing the CoQ_10_ content endogenously could be the next step to study in future.

Nevertheless, only a few studies have analysed the expression of mitochondrial genes during perinatal and early postnatal development [29,30]. Mitochondrial gene expression changes have been thoroughly reported in human foetal liver and skeletal muscle mtDNA expression and maintenance [9], but an adequate mapping of mitochondrial biogenesis in the perinatal period is still missing, with the exception of the β-F1-ATPase subunit, which was shown to be regulated at the transcriptional level during foetal development [31,32]. Our pilot study described the orchestration of mRNA expression of 20 genes important to ATPase biogenesis and mitochondrial oxidative metabolism in the liver and muscle tissue during rat perinatal development (*Rattus norvegicus* Berkenhout, 1769, var. alba; Wistar albino rat) [33]. We aimed to study changes in mitochondrial metabolism in perinatal period regardless of tissue type; therefore, skeletal muscle was chosen as a model tissue whose function stays generally unchanged throughout the mammalian perinatal period [33]. On the other hand, during the prenatal and perinatal periods, the liver plays several roles—haematopoiesis (already starting at F14 according to [34]), and fatty acid β-oxidation and other metabolic pathways, which considerably fluctuate during ontogenesis. Together with using the data obtained in human foetuses, we decided to apply a broad RNA microarray analysis to find specific interconnections of transcriptional regulators or activators with an emphasis on CoQ biosynthesis. We believe that these data may enable the identification of further key factors regulating mitochondrial activation, and also improve diagnostics of metabolic disorders and care in preterm newborns.

The aim of this project was to study gene expression, with a validation of selected genes involved in CoQ biosynthetic process, during foetal and early postnatal physiological development of *Rattus norvegicus, var. alba* (Wistar albino rat) in the liver and the skeletal muscle tissue and to compare the results with those found in humans.

## 2. Materials and Methods

The animals were kept and all rat tissues were obtained in the Department of Genetics of Model Diseases, Institute of Physiology of the Czech Academy of Sciences (CAS; Prague, Czech Republic). The animals had ad libitum access to standard rat chow and tap water. Rats were maintained on a twelve-hour diurnal cycle by automatic light switching. A total of 54 samples of liver and 35 samples of skeletal muscle (hind limb) from Wistar rat foetuses and neonates were obtained. A subset of 16-, 20- and 22-day-old foetuses (Foetal, hence F16, F20 and F22) and 1- and 4-day-old neonates (Postnatal, hence P1 and P4) were used for microarray analyses (4–5 replicates per time-point). For the rest of analyses, more time-points were analysed (between F16 and P18). All rat foetuses were delivered by Caesarean section after the sacrifice of pregnant Wistar rat mothers. Adult controls were collected (P90). All tissues were immediately snap-frozen in liquid nitrogen. Tissues were stored for further analyses at −80 °C. A total of 25 samples of human liver and 18 samples of human skeletal muscle were collected after the termination of pregnancy for genetic indications unrelated to mitochondrial deficiency. Tissues were obtained at autopsy less than 60 min post-mortem, immediately frozen, and stored at −80 °C. The foetal gestational age varied mainly from 13 to 29 weeks. The same set of liver and muscle samples was used for our previous analyses [9,35]. For CoQ_10_ content analyses in human, the control liver samples were obtained at autopsy of 7 children at ages between 0 and 15 months. A group of control muscle samples for CoQ_10_ content analyses was established from 9 “disease-free controls” at an age of 1–15 months.

### 2.1. RNA Preparation and cDNA Reverse Transcription

Tissue homogenates for mRNA analysis were prepared in TRI Reagent® solution (Molecular Research Center, Inc., Cincinnati, OH, USA) by ULTRA-TURRAX T8 homogenized (IKA, Staufen, Germany) following the manufacturer’s protocol. RNA was treated with a TURBO DNA-free™ Kit (Ambion, Austin, TX, USA). The quantity and quality of acquired total RNA was analysed by NanoDrop 1000 (Thermo Scientific, Wilmington, DE, USA) and Agilent Bioanalyzer 2100 (Agilent technologies, Waldbronn, Germany)—the RIN number was above 7 for all samples on a scale of 1–10. RNA samples were stored at −80 °C until use. cDNA was transcribed from 1000 ng of total RNA by Superscript III Reverse Transcriptase (Invitrogen, Carlsbad, CA, USA) with Oligo-dT primers (Promega, Madison, WI, USA). RT-minus control reactions without reverse transcriptase were also tested. cDNA was thawed only twice, and it was stored at −20 °C for no longer than two weeks to complete the qPCR analysis.

### 2.2. Quantitative PCR

Gene expression was validated by qPCR performed by TaqMan® Gene Expression Assay using TaqMan® probes FAM-MGB (Applied Biosystems, Waltham, MA, USA; *Psmb6* Rn00821581_g1, *Hprt* Rn01527840_m1, *Tbp* Rn01455646_m1, *Coq8a* Rn01415160_m1, *Coq2* Rn01450024_m1, *Coq3* Rn00569878_m1, *Coq4* Rn01758607_m1, *Coq5* Rn01500056_m1, *Coq6* Rn01517465_m1) in reactions containing 12.5 µL of 2 × TaqMan® Gene Expression Master Mix (Applied Biosystems, Bedford, MA, USA), 1.25 μL of the probe, 1 µL of the cDNA template, and water to bring the final volume up to 25 µL. Each cDNA sample was diluted immediately after reverse transcription, thus 1 μL of cDNA corresponded to 25 ng of total RNA used for reverse transcription. Thermal-cycling protocol was performed according to the manufacturer’s instructions. To assess the reaction efficiency of each probe (ranging at least between 90% and 110%), a calibration curve was prepared with pooled cDNA sample at dilutions 100, 50, 25, 12.5 and 6.25% (V/V). For the normalisation expression of genes, *Tbp* and *Psmb6* were used in the liver, while the expression of genes *Hprt* and *Psmb6* were used in the skeletal muscle, similarly to previously published results [9,33]. GenEx software was used for reaction efficiency and relative quantification of all genes. For each gene, the samples were analysed in duplicates, and the whole analysis was reproduced twice. Statistical analyses were performed in STATISTICA 12.0 (StatSoft, Tulsa, OK, USA) and R [36]. Illustrative expression curve profiles were obtained by least squares regression analysis. Constructed expression plots consisted of at least three samples of the same age quantified twice in duplicate. Each dot represents a mean value. Due to very limited amount of material available, *Coq7*, *Coq9* and *Ndufa9* transcripts were not validated by qPCR.

### 2.3. Protein Concentration

Protein concentration was measured by the method of Lowry [37].

### 2.4. Spectrophotometric Analysis of Mitochondrial Enzyme Activities

Samples of 10% liver homogenates (wet weight/V) were prepared in STE solution (10 mM Tris-HCl, pH 7.4; 250 mM sucrose; 1 mM EDTA; 1% fresh Protease Inhibitor Cocktail), 10% skeletal muscle homogenates were prepared in KTEA solution (150 mM KCl, 50 mM Tris-HCl, 2 mM EDTA, pH 7.5, freshly added aprotinin—2 µg/10mL KTEA). Tissues were homogenized by ULTRA-TURRAX T8 homogenizer (IKA, Staufen, Germany) and further smoothened on a Potter-Elvehjem homogenizer (Bellco glass, Vineland, NJ, USA). In human samples, the postnuclear supernatant (PNS) was isolated from the homogenate via centrifugation at 600× *g*, for 10 min, at 4 °C. The PNS was filtered through a nylon mesh. The mitochondria were sedimented by centrifugation of the PNS at 10,000× *g*, for 10 min, at 4 °C. The pellets were washed with the isolation medium, centrifuged again in the same conditions, and finally resuspended in the isolation medium at a protein concentration of approximately 20 mg/mL. In all tissue homogenates (rat) and isolated mitochondria (human), the activities of electron transport chain (ETC) complexes were measured at 37 °C using a Shimadzu 2401 UV–Vis spectrophotometer (Shimadzu Corporation, Kyoto, Japan): NADH:coenzyme Q_10_ oxidoreductase (complex I), succinate:coenzyme Q_10_ oxidoreductase (complex II), coenzyme Q_10_:cytochrome c oxidoreductase (complex III), NADH:cytochrome c oxidoreductase (complex I–III), succinate:cytochrome c oxidoreductase (complex II–III) was assayed according to [38] and cytochrome c oxidase (complex IV) according to [39]. Citrate synthase (CS) was measured according to [38]. For one assay, a sample representing approximately 20 µg of homogenate or mitochondrial protein was used.

To measure the activities of complexes I and I–III, the sample was incubated for 3 min in distilled water to disrupt the mitochondrial membranes. The rotenone-sensitive complex I activity was then measured in 1 mL of assay medium (50 mM Tris-HCl, pH 8.1, 2.5 mg/mL BSA, 50 µM decylubiquinone, 0.3 mM KCN, and 0.1 mM NADH with and without 3 µM rotenone) based on the decrease in absorbance at 340 nm due to NADH oxidation (ε = 6.22 mM^−1^cm^−1^).

The rotenone-sensitive complex I–III activity was determined by incubating the sample in 1 mL of assay medium (50 mM Tris-HCl, pH 8.1, 2.5 mg/mL BSA, 40 mM cytochrome c, 2 mM KCN, and 0.1 mM NADH with and without 3 µM rotenone) and measuring the increase in absorbance at 550 nm (ε = 19.6 mM^−1^cm^−1^) due to the reduction in cytochrome c.

Complex II activity (succinate-DCPIP oxidoreductase) was determined by incubating the sample in 1 mL of assay medium (10 mM potassium phosphate, pH 7.8, 2 mM EDTA, 1 mg/mL BSA, 0.3 mM KCN, 10 mM succinate, 3 µM rotenone, 0.2 mM ATP, 80 µM DCPIP, 1 µM antimycin, and 50 µM decylubiquinone) and measuring the decrease in absorbance at 600 nm due to the reduction in DCPIP (ε = 20.1 mM^−1^cm^−1^).

Complex II–III activity was determined by incubating the sample in 1 mL of assay medium (50 mM potassium phosphate, pH 7.8, 2 mM EDTA, 1 mg/mL BSA, 0.3 mM KCN, 10 mM succinate, 3 µM rotenone, 0.2 mM ATP and 40 µM cytochrome c) and measuring the increase in absorbance at 550 nm (ε = 19.6 mM^−1^cm^−1^).

Complex III activity was determined by incubating the sample in 1 mL of assay medium (50 mM KPi pH 7.8, 2 mM EDTA, 1 mg/mL BSA, 0.3 mM KCN, 50 µM cytochrome c, and 50 µM ubiquinol) and measuring the increase in absorbance at 550 nm (ε =19.6 mM^−1^cm^−1^).

Complex IV activity was determined in isolated mitochondria by incubating the sample in 1 mL of assay medium (40 mM potassium phosphate, pH 7.0, 1 mg/mL BSA, 25 µM reduced cytochrome c, and 2.5 mM n-dodecyl-β-D-maltoside) and measuring the oxidation of reduced cytochrome c (II)—the decrease in absorbance at 550 nm (ε = 19.6 mM^−1^cm^−1^).

CS activity was determined using a mixture containing 100 mM Tris-HCl, pH 8.1, 0.1 mM DTNB (5,5’-dithio-bis(2-nitrobenzoic acid, 2.5 mM n-dodecyl-β-D-maltoside), the sample, 0.5 mM acetyl coenzyme A, and 0.5 mM oxaloacetate. The activity was measured at 412 nm (ε = 13.6 mM^−1^cm^−1^). For calculation of the final background activity, the activity without oxaloacetate was subtracted.

The activities were expressed as nmol of substrate converted per minute and normalised to the protein content in the reaction (nmol/min.mg protein). All chemicals were purchased from Sigma-Aldrich (Saint Louis, MO, USA).

### 2.5. CoQ_9_ and CoQ_10_ Content

Total CoQ coenzyme content was determined according to [40], with minor modifications. Tissue homogenate (100 µL 10% homogenate + 100 µL water) was vortexed with the addition of 50 µL 1,4-benzoquinone (2 mg/mL; Sigma-Aldrich, Saint Louis, MO, USA) and left for 10 min at room temperature. Then, 1 mL of propan-1-ol was added and the mixture was vortexed properly. Samples were centrifuged (26,000× *g*, 20 min, 4 °C) and the acquired supernatant was used for HPLC measurement. The amount of CoQ was measured using HPLC Pharmacia on reverse-phase Supercosil LC 18 and LC 18S (Supelco) column with an in-line filter (Upchurch Scientific), mobile phase ethanol:methanol, 7:3, 1 mL/min, detection at 275 nm using standard CoQ_10_ solution (TANAKA, Tokyo, Japan) [40,41] and CoQ_9_ solution (Sigma-Aldrich, Saint Louis, MO, USA). The CoQ content in the tissue homogenate was expressed in pmol per milligram of protein.

### 2.6. cDNA Microarray Performance and its Subsequent Data Analysis

Genome-wide mRNA expression microarray analysis was conducted using the GeneChip Rat Gene 1.0 ST Arrays (Affymetrix, Santa Clara, CA, USA). The RNA which was used for this analysis was purified from both tissues at five developmental points (16th, 20th, and 22nd foetal (F16, F20, F22) and first and fourth postnatal (P1, P4)) using an RNeasy Mini Kit (QIAGEN, Hilden, Germany). The generation of labelled cDNA, hybridizations, and microarray scanning were performed by The Centre for Applied Genomics, The Hospital for Sick Children, Toronto, Canada. The data were assessed for quality and subjected to robust multi-array averaging (RMA) normalisation (Affymetrix expression console; Affymetrix, Santa Clara, CA, USA) in R [36] (topGO library) [42,43,44]. The microarray datasets supporting this publication have been deposited in NCBI’s Gene Expression Omnibus [45] and are available there through GEO Series accession number GSE131012 (https://www.ncbi.nlm.nih.gov/geo/query/acc.cgi?acc=GSE131012 accessed on 5 May 2021) [reviewers token: obozymwetfezxip]. This dataset also includes F18 (skeletal muscle) and adult (liver) samples, but these data points were excluded from the bioinformatic analyses stated in the manuscript and we did not use them to make any conclusions. As was previously published [46], the application of multiple testing adjustments to *p*-values may result in a loss of significant mitochondria-related genes with subtle changes in the expression. The only *p*-value cut-off (*p* ≤ 0.05) was applied after gene ontology analysis (no fold change restrictions). Heatmaps of gene expression were created using the programs GenEx (MultiD Analyzes AB, Göteborg, Sweden) and R [36]. Transposed data were auto-scaled, and Ward’s algorithm was chosen as a clustering method according to Pearson’s correlation coefficient distance. Data were also analysed in Short Time-Series Expression Miner—STEM 1.3.8 [47]—and the factor change was set to 2; hence, data were clustered to 81 types of profiles. We used also TIGR MeV 4.9 (betr package) [48]. Results were considered significant when the corresponding *p* ≤ 0.05.

### 2.7. Data Analysis and Statistics

Statistical analyses of CoQ_9_ and CoQ_10_ content, enzyme activities, and qPCR were performed in STATISTICA 12.0 (StatSoft, Tulsa, OK, USA) and R (“R Core Team” URL: http://www.r-project.org/ accessed on 3 May 2021). Illustrative expression curve profiles were obtained by least squares regression analysis. Results were considered significant when the corresponding *p* ≤ 0.05.

For Pearson’s correlation-based hierarchical clustering of genes, data were transposed and auto-scaled in GenEx (MultiD Analyzes AB, Göteborg, Sweden). Ward’s algorithm was chosen as a clustering method according to Pearson’s correlation coefficient distance to construct the heatmap in R (“R Core Team” URL: http://www.r-project.org/ accessed on 3 May 2021).

## 3. Results

### 3.1. CoQ_9_ and CoQ_10_ Content

**Figure 1:**
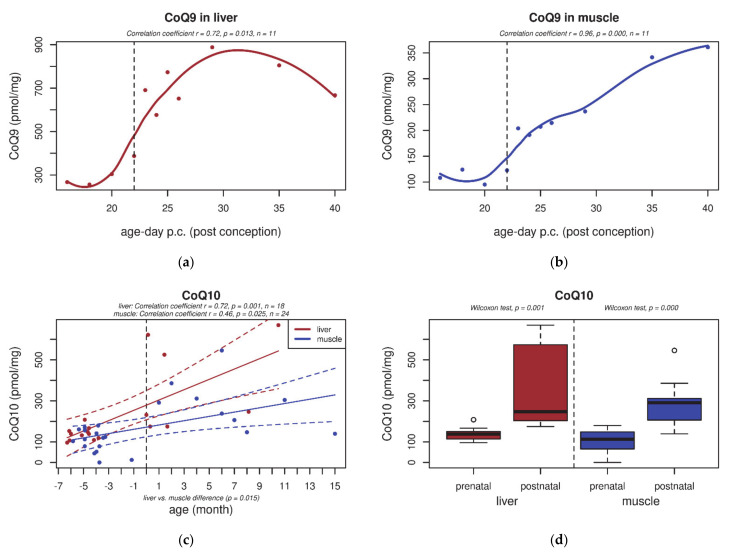
Total CoQ_9_ and CoQ_10_ content (pmol/mg protein) in rat and human tissues, respectively, during early development. (**a**,**b**) In both rat liver and skeletal muscle tissue, CoQ_9_ content increased significantly after birth (*p* ≤ 0.05); (**c**,**d**) in rats, this was not observed for CoQ_10_ content (data not shown). Similarly, CoQ_10_ content in human tissue was significantly increased after birth (*p* ≤ 0.05). The black dashed line indicates the last foetal day/birth. The red and blue dashed lines indicate 95% confidence intervals in the liver and skeletal muscle, respectively.

**Figure 2:**
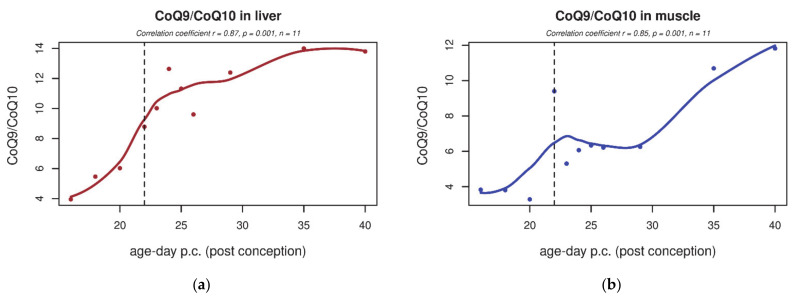
CoQ_9_/CoQ_10_ ratio in rat tissues during early development. (**a**,**b**) In both rat liver and skeletal muscle tissue, CoQ_9_/CoQ_10_ ratio is significantly higher after birth (*p* ≤ 0.05). The dashed line indicates the last foetal day/birth.

In both rat tissues, the content of total CoQ_9_ increased after birth (Figure 1a,b). Compared to stage F16, postnatal CoQ_9_ content increased by 100% or more in the liver and by about 80% in skeletal muscle. The CoQ_10_ content remained very low (about 15% of the neonatal CoQ_9_ content, data not shown). The CoQ_9_/CoQ_10_ ratio in rats was significantly higher in both tissues after birth (Figure 2a,b). Similarly, the CoQ_10_ content in human tissues also significantly increased in newborns compared to premature human foetuses (Figure 1c,d).

### 3.2. Electron Transport Chain (ETC) Enzyme Activities

**Figure 3:**
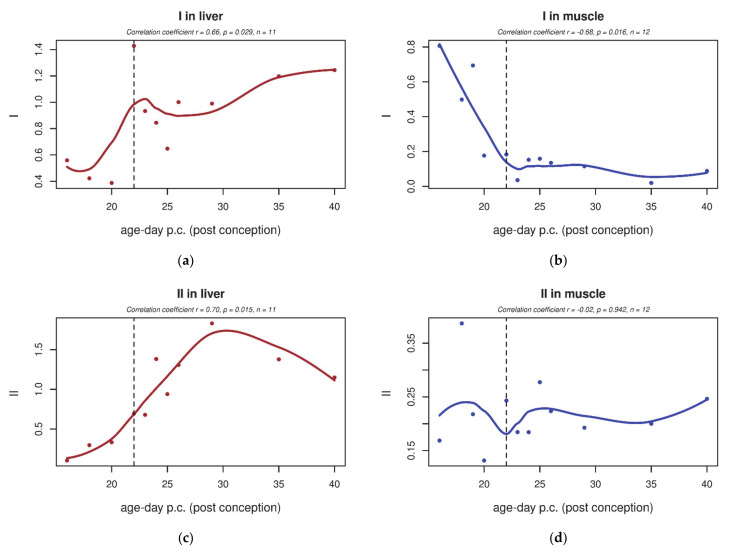

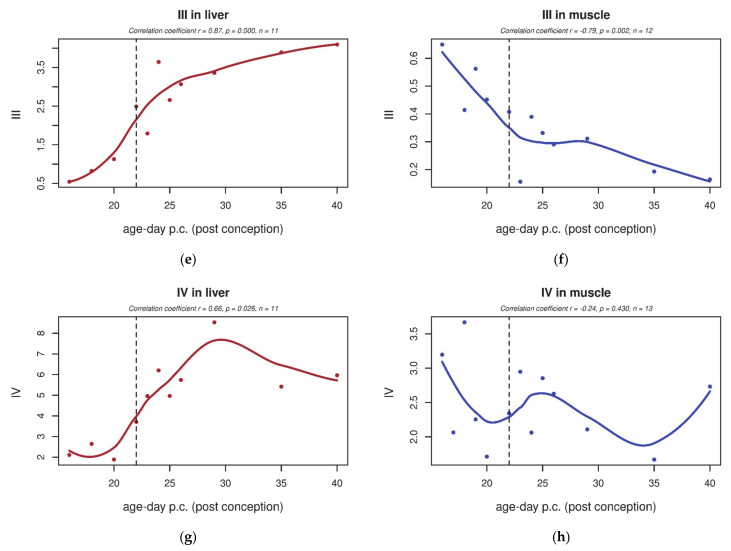
Tissue-specific changes in electron transport chain (ETC) complexes’ activities in rat liver and skeletal muscle during early development. (**a**,**c**,**e**) In the liver, enzyme activities of ETC complexes significantly increased after birth. (**b**,**d**,**f**) In skeletal muscle, only the activities of complexes I (**b**) and III (**f**) changed, decreasing significantly after birth (*p* ≤ 0.05). (**g**) In the liver, complex IV activity significantly increased after birth (*p* ≤ 0.05). (**h**) In skeletal muscle, complex IV activity did not significantly change after birth. Enzyme activities normalised to citrate synthase are shown. The dashed line indicates the last foetal day/birth. Enzyme activities normalised to citrate synthase are shown. The dashed line indicates the last foetal day/birth. I—NADH:coenzyme Q_10_ oxidoreductase, II—succinate:coenzyme Q_10_ oxidoreductase, III—Coenzyme Q_10_:cytochrome c oxidoreductase, IV—cytochrome c oxidase.

**Figure 4:**
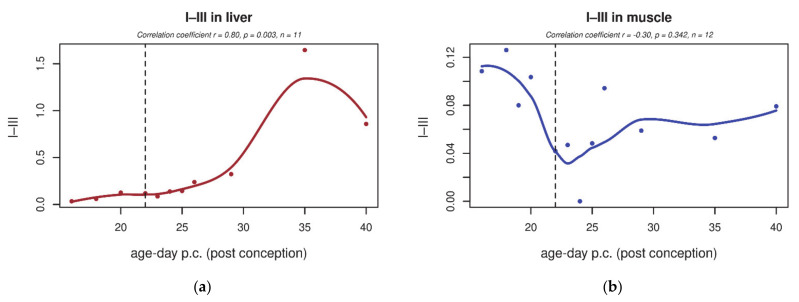

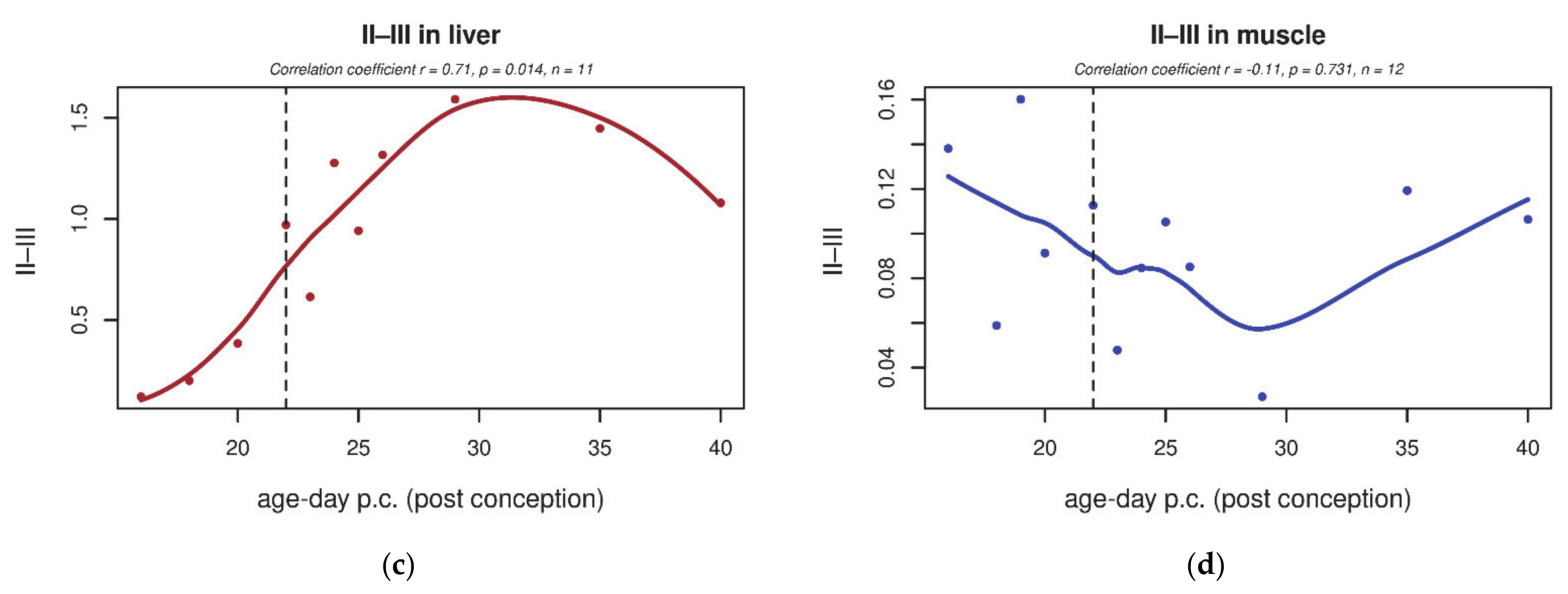
Tissue-specific changes in electron transport chain (ETC) complexes’ coupled activities in rat liver and skeletal muscle during early development. (**a**,**c**) In the liver, coupled activities of complexes I–III and II–III significantly increased after birth (*p* < 0.05). (**b**,**d**) Contrary to observations in the liver, a significant increase in activities of complexes I–III and II–III was absent in skeletal muscle. Enzyme activities normalised to citrate synthase are shown. The dashed line indicates the last foetal day/birth. I–III—NADH:cytochrome c oxidoreductase, II–III—succinate:cytochrome c oxidoreductase.

Changes in ETC enzyme activities were profound in the rat liver: after birth, the ETC activity increased globally, including complexes I, III and IV (Figure 3a,e,g). A minor increase was also detected in complex II activity (Figure 3c). Coenzyme Q transfers electrons in ETC from complexes I and II to complex III; therefore, we were also interested in I–III and II–III coupled activity measurements (Figure 4a,c), both of which increased significantly in the liver. In contrast to the liver, in skeletal muscle, despite the similar increase in CoQ_9_ content, we saw a slightly decreased activity of complex I and III (Figure 3b,f). In skeletal muscle, no significant changes were detected in isolated complex II and IV activity (Figure 3d,h) or in I–III and II–III coupled activity (Figure 4b,d).

**Figure A1:**
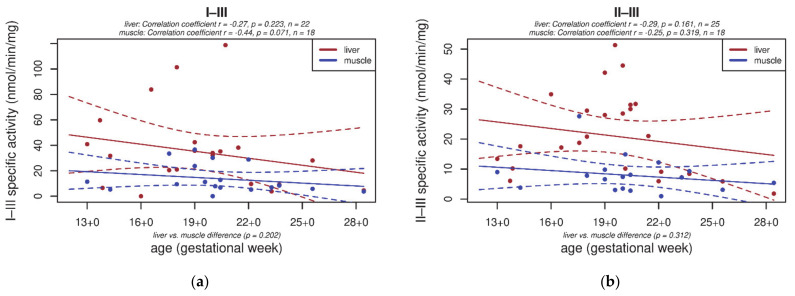
Tissue-specific changes in electron transport chain (ETC) complexes’ coupled activities in foetal human liver and skeletal muscle. In the liver (red) and in skeletal muscle (blue), coupled activities of complexes I–III (**a**) and II–III (**b**) were decreasing between the 13th and 28th week of gestation, but not significantly. Enzyme activities are shown as specific activity (nmol/min/mg). Statistical analyses were performed in STATISTICA 12.0 (StatSoft, Tulsa, OK, USA) and R (“R Core Team” URL: http://www.r-project.org/ accessed on 3 May 2021). The dashed line indicates 95% confidence level. I–III—NADH:cytochrome c oxidoreductase, II–III—succinate:cytochrome c oxidoreductase.

In human tissues, the activities of complexes I–III and II–III (Figure A1a,b) decreased between the 13th and 28th week of gestation (see Appendix A). This prenatal decreasing trend is similar to the findings in rat skeletal muscle (although not significant).

### 3.3. Microarray Analysis

Microarray data showed that 54.28% of genes expressed in the liver had an altered expression at least at one point of the time series (“active genes”). In skeletal muscle, 63.81% of the genes expressed were identified as “active”. Regarding mitochondrial genes, 82.56% were “active” in the liver vs. 87.26% in skeletal muscle. Interestingly, 70% of these “active” mitochondrial genes in the liver had been differentially expressed, even prenatally (comparing period F16–F22). No GO Terms were identified as significantly changed in the intervals F22–P1 and P1–P4 in the liver.

Among the biological processes that did significantly change in both tissues (in the interval F16–P4), four GO Terms were identified. “Amino-acid betaine metabolic process” (12 genes) and “endocytosis” (335 genes) were significantly upregulated, “mitochondrial genome maintenance” (25 genes) decreased in the liver and skeletal muscle. “Regulation of peptidyl-serine phosphorylation” (86 genes) increased in the liver, but decreased in skeletal muscle (selected genes are listed Table 1). In the liver, the analysis of the set of significantly changed mitochondrial genes revealed that ten GO Terms were significantly enriched (Table 2). No such enrichment in any GO Term was identified in skeletal muscle. The Supplementary Material contains the tables showing the significantly changed GO categories for every mentioned profile and/or the tables of gene symbols co-clustered in this analysis (Table S1).

**Figure 5:**
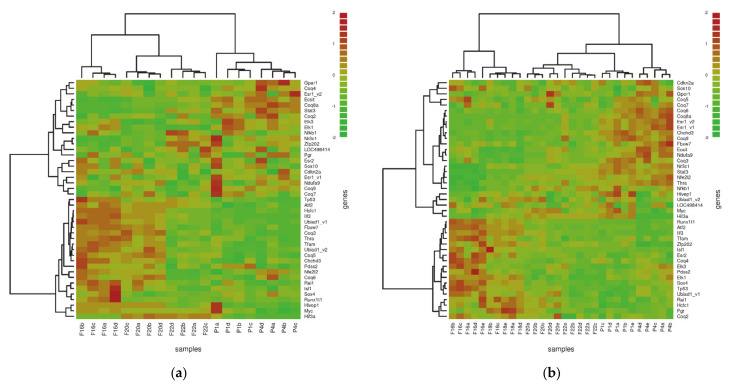
Pearson’s correlation-based hierarchical clustering of genes annotated for CoQ biosynthesis and mitochondrial transcription activity regulators. Data were auto-scaled. (**a**) Heatmap shows that in the liver, *Coq2*, *Coq4*, *Coq7* and *Coq9* expression was increased after birth, as was the *Coq8a* expression. *Coq3* and *Coq5* expression was decreased. (**b**) This orchestration, contrary to the liver, was not observed in skeletal muscle. Interestingly, in skeletal muscle, *Coq3*, *Coq5*, *Coq6*, *Coq7*, *Coq9* and *Ndufa9* co-clustered with the *Coq8a* transcript. In both tissues, *Tfam* expression decreased throughout the observed period, whereas oestrogen-related receptor (*Esr1*) expression increased. For *Esr1* and *Ubiad1*, there were two probes on the array (marked with _**v1** or _**v2**). F—Foetal day, P—Postnatal day.

Among the “mitochondrial” genes, we searched for those connected with CoQ biosynthesis (Table 3). In the liver, *Coq3*, *Coq5* decreased, while *Coq9* increased significantly as well as *Coq8a* (formerly *Adck3* or *Cabc1*). In skeletal muscle, *Coq4* decreased significantly. In both tissues, *Coq8a* and *Ndufa9* increased several-fold (four- and three-fold, respectively. Pearson’s correlation-based hierarchical clustering revealed that in the liver, the expression of *Coq2*, *Coq4*, *Coq7* and *Coq9* increased after birth (Figure 5a). In skeletal muscle, this was not found as a general trend, but the expression profiles of *Coq3*, *Coq5*, *Coq6*, *Coq7*, *Coq9* and *Ndufa9* correlated with the increasing content of *Coq8a* after birth (Figure 5b). Microarray data were also analysed in STEM [47], appropriate for the time series data. In the liver, we identified four clusters with a significantly enriched number of genes, correlating with 10 distinct profile types. Four of them were descending with age (see Appendix A, Table A1), while the rest were ascending. In skeletal muscle, we identified six clusters with 10 profiles again (three of them descending), possibly playing a role in the expression switch during the perinatal phase. The list of all genes clustered in Table A1 is attached in the Supplementary Material (Table S1).

**Figure A2:**
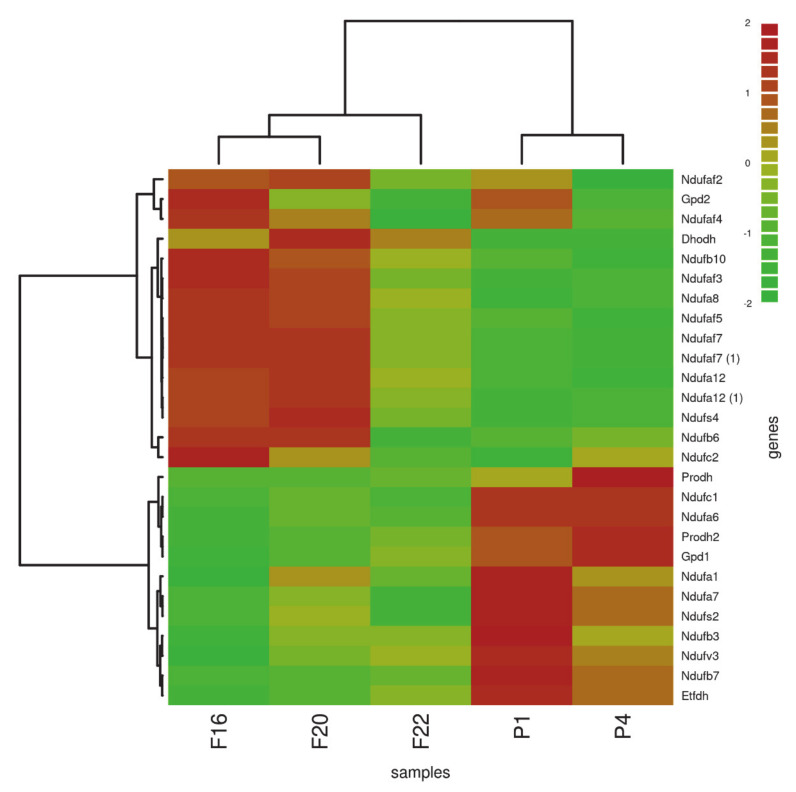
Liver-specific changes in the expression of genes coding selected enzymes involved in CoQ metabolism and function. Changes in expression of genes coding complex I (including assembly factors; *Nduf*), proline dehydrogenase (*Prodh*), glycerol-3-phosphate dehydrogenase (*Gpd*), electron-transferring-flavoprotein dehydrogenase (*Etf*) and dihydroorotate dehydrogenase (quinone; *Dhodh*) were analysed in rat tissues. In the rat liver, the expression of all genes included in the heatmap changed significantly (*p* ≤ 0.05, betr package). Average values from 4 biological replicates were used. Data were transposed and auto-scaled in GenEx (MultiD Analyzes AB, Göteborg, Sweden). Ward’s algorithm was chosen as a clustering method according to Pearson’s correlation coefficient distance to construct the heatmap in R ("R Core Team" URL: http://www.r-project.org/ accessed on 3 May 2021). For *Ndufaf7* and *Ndufa12*, there were two probes on the array (marked with (1)). In skeletal muscle, only expression of *Ndufaf7* and *Ndufb6* was significantly decreased after birth; no other changes were detected among genes clustered in Figure A2 (not shown). F—Foetal day, P—Postnatal day.

CoQ is a cofactor of several enzymes; hence, we also clustered the genes that code for complex I (including assembly factors), proline dehydrogenase, glycerol-3-phosphate dehydrogenase, electron-transferring-flavoprotein dehydrogenase and dihydroorotate dehydrogenase (quinone) (Appendix A, Figure A2). Some of these genes were found to be significantly enriched according to GO analysis (see Table 2)—*Etfa*, *Etfb*, *Etfdh*, *Ndufaf7*, *Gpd1 and Gpd2*).

**Figure 6:**
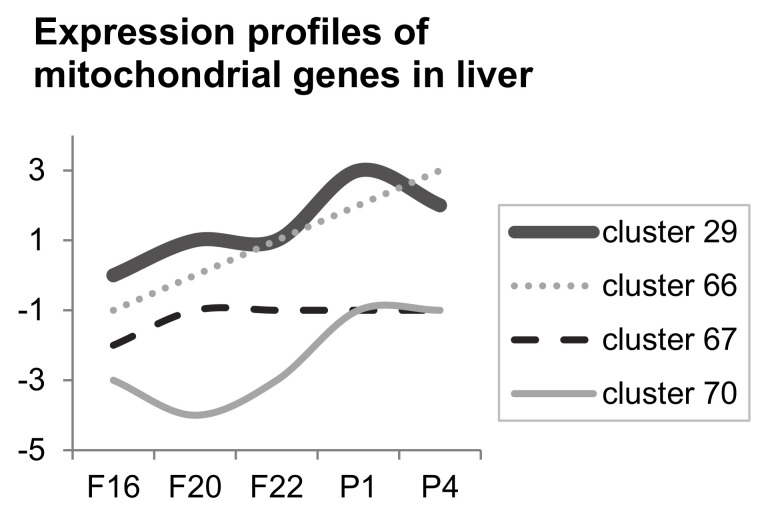
Four types of expression profiles significantly enriched in the rat liver (164 mitochondrial genes). A total of 1546 mitochondrial genes were analysed in STEM. Among 81 possible clusters, clusters named 29, 66, 67 and 70 were found to be significantly enriched in the liver (total 164 genes correlated with these four expression profile types—for gene table, see Table S1). No such enriched clusters were found in skeletal muscle, although GO “mitochondrion” GO:0005739 was found to be significant in both tissues per se. Analysis was performed in STEM [47].

From the STEM results, we analysed the genes annotated with the GO term “mitochondrion” (GO:0005739), identifying 164 genes to be co-clustered in profiles with significantly more genes than expected in the liver. The expression changes in these genes resembled the following profiles: cluster 29—(0.0, 1.0, 1.0, 3.0, 2.0), cluster 66—(0.0, 1.0, 2.0, 3.0, 4.0), cluster 67—(0.0, 1.0, 1.0, 1.0, 1.0) and cluster 70—(0.0, −1.0, 1.0, 2.0, 2.0) (see the profiles of these clusters during the observed period in Figure 6). Only 12 of these genes decreased their expression during development, and the decrease was observed prenatally (between F16 and F22). All co-clustered genes showed a higher expression after birth than at F16. In skeletal muscle, the same analysis revealed the GO:0005739 “mitochondrion” to be significantly changed per se, but no specific profiles were identified. The list of genes clustered to clusters 29, 66, 67 and 70 is attached in the Supplementary Material (Table S1).

Microarray data for genes annotated in GO:0005739 were analysed in TIGR MeV, betr package. This analysis revealed that, out of 1546 probes, 1091 were significantly changed in the liver (71%, Tables S2 and S3), while 821 were significantly changed in skeletal muscle (53%, Tables S2 and S3). To find out which genes are perinatally important regardless of tissue origin, genes were weight-ranked according to the fold change. Only a *p*-value cut-off without any fold change cut-off was applied, which is suitable for mitochondrial genes [46]. We obtained 248 unique genes that were significantly changed in both tissues regardless of tissue origin (Table S4). The most differentially expressed gene was *Pdk4*, pyruvate dehydrogenase kinase, isozyme 4.

### 3.4. qPCR

**Figure 7:**
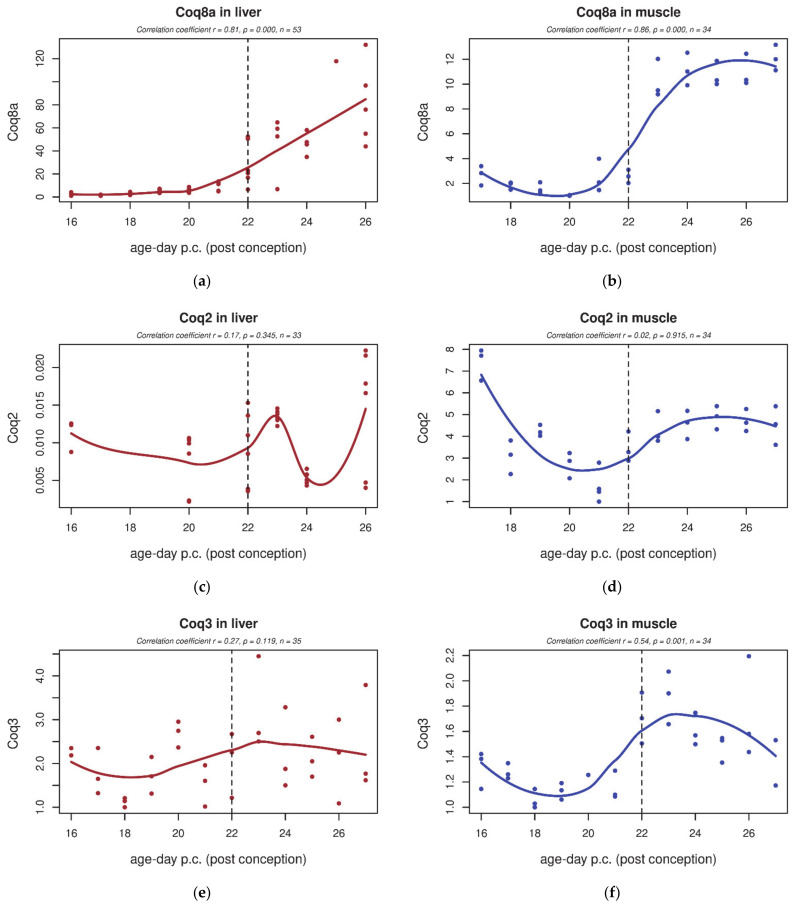
*Coq8a*, *Coq2*, and *Coq3* expression in rat tissues during early development. (**a**,**c**,**e**) In the rat liver, we detected significantly increased *Coq8a* transcripts after birth. *Coq2* and *Coq3* were unchanged (**c**,**e**). (**b**,**d**,**f**) In skeletal muscle, postnatal *Coq8a* was significantly increased (**b**). Genes *Coq2* and *Coq3* did not show any significant trend during perinatal development. The dashed line indicates the last foetal day/birth.

**Figure 8:**
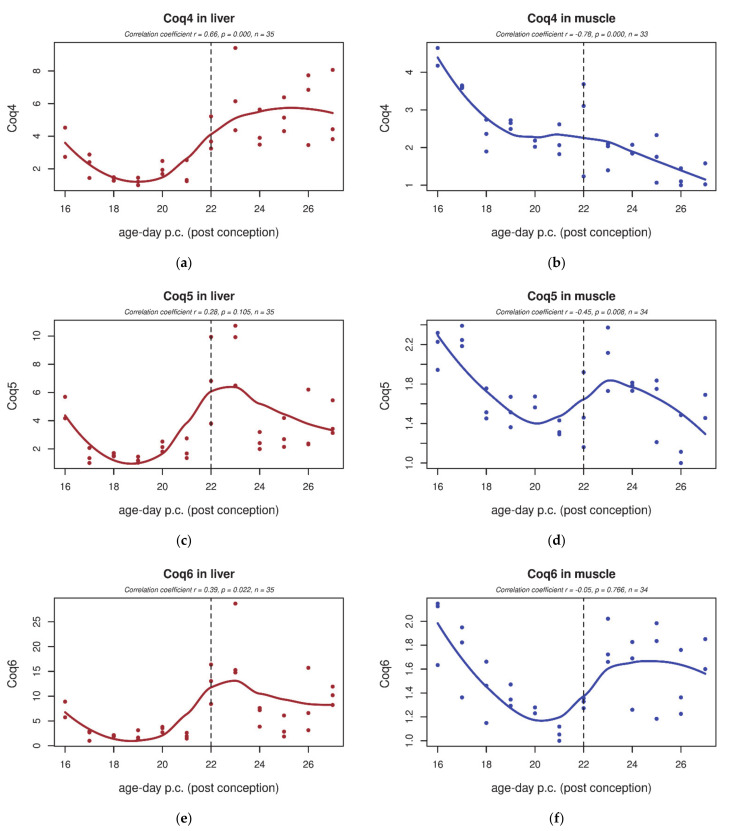
*Coq4*, *Coq5* and *Coq6* expression in rat tissues during early development. (**a**) In the rat liver, we detected that the *Coq4* transcript is significantly increased after birth. (**c**,**e**) *Coq5* and *Coq6* showed a transiently (but not significantly) increased expression around days F22 and P1. (**b**) In skeletal muscle, *Coq4* showed a decreasing tendency throughout the whole studied period. (**d**,**f**) Genes *Coq5* and *Coq6* did not show any significant trend during perinatal development. The dashed line indicates the last foetal day/birth.

Gene expression of selected genes involved in CoQ biosynthesis was validated by qPCR in a larger set of samples throughout the development of both tissues (*Coq2–6*, *Coq8a*) (Figure 7 and Figure 8). In the liver, we detected that both *Coq4* and *Coq8a* transcripts were significantly increased after birth (Figure 7a and Figure 8a). *Coq5* and *Coq6* showed a transiently increased expression around days F22 and P1, but without significance (Figure 8c,e). *Coq2* and *Coq3* were unchanged (Figure 7c,e). In skeletal muscle, *Coq3* and *Coq8a* significantly increased postnatally (four-fold increase in *Coq8a* compared to prenatal expression; Figure 7b,f). *Coq4* showed a decreasing tendency throughout the whole studied period (Figure 8b,d). Other genes (*Coq2*, *Coq6*) did not show any significant trend during perinatal development (Figure 7d and Figure 8f).

## 4. Discussion

Mitochondrial biogenesis was studied extensively in a number of models, including mice and rats, but mostly postnatally [5,30,31,49,50,51]. Later, selected proteins were also studied in human foetal liver and skeletal muscle [9,35]. This work builds upon our pilot study [33] using a broad microarray dataset from the perinatal period to illustrate the orchestration of the mitochondrial metabolic response at the transcriptional level. Our data confirm the skeletal muscle as a model tissue whose development is rather gradual throughout the mammalian perinatal development. On the other hand, the microarray analysis emphasized significant changes in a number of mitochondrial genes which were significantly changed in the liver during the perinatal period (Table 1).

At the CoQ content level, findings in both the rat model and human tissues showed a similar trend, even though there are some limitations of using human autopsies from foetuses with genetic indications: the total CoQ content is low prenatally, significantly increasing after birth in both the liver and skeletal muscle (Figure 1). A significant increase in CoQ_10_ was already shown in the plasma of newborns [18], reaching normal adult levels within the first month of life in clinically “stable” infants. 

As indicated by our microarray data, the expression changes during the studied period are enormous. Of all analysed genes expressed in the liver and muscle, 54.28% and 63.81% were identified as “active”, respectively, i.e., showing an altered expression at least at one point of the time series. Among these, 6–15% of genes are enriched with an organ-specific phenotype, as Cardoso-Moreira et al. identified earlier [52]. The increased CoQ_9_ level and OXPHOS activities in the liver after birth led us to concentrate on the mitochondrial metabolism (1119 and 827 out of 1546 mitochondrial genes are “active” in the liver and muscle, respectively). In agreement with our previous conclusions [33], the critical changes in rat liver occur prenatally between days F16 and F22, at least two days before birth. This means that, at the transcription level, rat liver tissue is already differentiated and mature enough to undergo transition to extra-uterine conditions by day F22. This conclusion is somewhat different from that of Hurley et al., who described a later significant change in about one-half of mitochondrial genes in the rat liver, across the F21–P7 time-course [53]. These authors claimed that genes encoding components of the respiratory chain showed no coordinated regulation, but they investigated a single foetal time point (E21; equal to our F21) and focused their work more on the early rat liver postnatal development (30 min, 4 h, 12 h, 24 h and 7 days after birth).

Although we did not find the GO term “ubiquinone biosynthetic process” (GO:0006744) to be significantly changed in our study, we still validated the gene expression of selected genes using qPCR. Indeed, a microarray-based study of fibroblasts derived from patients diagnosed with primary CoQ_10_ deficiency similarly detected no changes in the overall GO term “ubiquinone biosynthetic process” (GO:0006744), although demonstrated several fold changes in the relative count of single transcripts [54]. Considering the individual genes, *COQ8A* (here, we showed a four-fold increase in expression after birth) together with *COQ8B* have been predicted as regulators of CoQ biosynthesis in yeast and humans [55,56]. Our data in rats show an increase in *Coq2*, *Coq4*, *Coq7* and *Coq9* expression in the liver after birth (Figure 5a) and that of *Coq3*, *Coq5–7*, *Coq9* and *Ndufa9* in skeletal muscle (Figure 5b). Cullen et al. showed that *COQ8A* interacts with *COQ3*, *COQ5*, *COQ7* and *COQ9* in HeLa cells [57,58]. This correlates with another study, which identified PTC7, which was not annotated on the microarray in our study, to be a regulator of CoQ in human HeLa and SH-SY5Y cells [59]. These authors found that the expression of *COQ5*, *COQ6*, *COQ8* and *COQ9* increases together with that of *PTC7* in starving cells, whereas the expression of *COQ1*, *COQ2*, *COQ3*, *COQ4* and *COQ7* does not [59].

In yeast, another regulator of the CoQ biosynthetic machinery was identified in PUF3, a 3’-UTR mRNA and miRNA binding protein [60]. Lapointe et al. claimed that the CoQ biosynthetic complex can be disrupted when *Pum1* and *Pum2*, the rat homologs of *PUF3*, show a lower expression, while *Coq5* shows a higher expression. We observed that both *Pum1* and *Pum2* expression decreases significantly during the F16–P4 period (data not shown). However, we were not able to find an increase in *Coq5* expression as significant by qPCR. The 3’-UTR mRNA or miRNA binding regulators such as *Pum1* and *Pum2* target the expression of hundreds of downstream components in oxidative stress response and cell cycle regulation at the post-transcriptional level [61,62]. This regulatory mechanism was also shown for miR-127-5p, a key driver of β-ATPase subunit translation [32]. Moreover, even at the post-translational level, the interaction partners regulating the final response were identified in yeast and human cells: OCT1 and protein phosphatase PTC7 [59,63]. We found that the postnatal expression of both these partners significantly increased in the rat liver, but not in rat muscle (data not shown). However, all these steps of gene expression regulation seem to be critical in mitochondrial pathology occurrence in patients [64,65].

More than 70 patients suffering from primary CoQ_10_ deficiency were described in the literature, and novel cases appear every year [66,67,68]. However, up to 123,789 patients were predicted according to the prevalence of homozygous and compound heterozygous afflicted individuals worldwide (27,321 patients carrying a mutation in *COQ8A*) [69]. This illustrates how many undiagnosed cases we might be missing.

Importantly for broader research, mitochondrial respiration is not the only function for which CoQ is essential. For instance, the CoQ_9_-deficient murine knockout model (*Mclk1* gene), which arrests development at midgestation, suggests that a properly assembled CoQ-biosynthesis complex is generally necessary for vertebrate embryonic development [70], although some genes involved in “ubiquinone biosynthetic process” are non-essential, and *Pdss2*, *Coq8a* or *Coq9* mutant mouse models do not show prenatal lethality [55,71,72]. This is why the microarray data in this publication, a total gene count of 16,557 RefSeq (Entrez), have been deposited in NCBI’s Gene Expression Omnibus [45] to be available for the broad scientific community through the GEO Series accession number GSE131012 (https://www.ncbi.nlm.nih.gov/geo/query/acc.cgi?acc=GSE131012 accessed on 5 May 2021) [reviewers token: obozymwetfezxip].

## 5. Conclusions

The perinatal metabolic switch is an extremely complex process, associated with tissue proliferation and differentiation together with a rapid oxidative stress response. Moreover, this process proceeds on multiple levels—transcription, mRNA stability, post-translational modifications, etc. We used various techniques to highlight the orchestration of the perinatal metabolic switch from glycolytic to mitochondrial metabolism and compared the results with those obtained from human samples, both in the liver and skeletal muscle. The most differentially expressed genes in both tissues were *Pdk4* (pyruvate dehydrogenase kinase, isozyme 4) and, from those involved in the CoQ biosynthetic process, *Coq8a* (atypical kinase). We believe that these data could serve as a suitable background for future research, in particular for finding key factors regulating mitochondrial metabolism and the preparation of the foetus for the transition to extra-uterine conditions.

Finally, all the microarray data in this publication have been deposited in NCBI’s GEO, are available there through the GEO Series accession number GSE131012, which might be helpful for further studies by the whole scientific community.

## Figures and Tables

**Table 1:** Biological processes (GO Terms) that were significantly changed in the rat liver and skeletal muscle. Trend L/M shows decreasing (negative) or increasing (positive) expression tendency in the liver (L) and skeletal muscle (M) during the perinatal period—16th foetal day to the 4th postnatal day. Only the most important genes (25) are shown per each GO Term. (FDR ≤ 0.05).

GO Term	Trend L/M	Genes	Total Count
Mitochondrial genome maintenance	−0.003 (L) −0.017 (M)	*Peo1*, *Polg*, *Mrpl17*, *Tp53*, *Lig3*, *Dnaja3*, *Flcn*, *Opa1*, *Parp1*, *Akt3*, *Ppargc1a*, *Primpol*, *Tk2*, *Tfam*, *Slc25a16*, *Dna2*, *Mgme1*, *Stoml2*, *Slc25a33*, *Rnaseh1*, *Rrm2b*, *Pif1*, *Pid1*, *Pif1*, *Mef2a*	25
Amino-acid betaine metabolic process	0.208 (L) 0.03 (M)	*Cpt1a*, *Cpt1c*, *Por*, *Chdh*, *Aldh7a1*, *Dmgdh*, *Acadm*, *Tmlhe*, *Crat*, *Bbox1*, *Crot*, *Acadl*	12
Endocytosis	0.022 (L) 0.021 (M)	*Mex3b*, *Unc119*, *Cd9*, *Sfrp4*, *Cd36*, *Mapkapk3*, *Tinagl1*, *Wnt5a*, *Gsn*, *Cav2*, *Cav2*, *Eef2k*, *Ubqln2*, *Pycard*, *Cav1*, *Cdc7*, *Gas6*, *Mrc1*, *Ap2b1*, *Cd163*, *Tgfbr2*, *Dnm2*, *Tub*, *Nlgn3*, *Enpp3* and others	335
Regulation of peptidyl–serine phosphorylation	0.009 (L) −0.001 (M)	*Dmd*, *Wnt5a*, *Cav1*, *Gas6*, *Pde4d*, *Nos1*, *Sfrp2*, *Akt2*, *Fnip1*, *Prkd1*, *Cd44*, *Gpd1l*, *Hrc*, *Rassf2*, *Gsk3b*, *Mif*, *Ntf3*, *Tgfb1*, *Txn1*, *Bcl2*, *Gsk3a*, *Camk1*, *Arrb2*, *Ogt*, *Gfra2* and others	86

**Table 2:** Biological processes (GO Terms) connected with “mitochondrial metabolism” that were significantly changed in the rat liver. Several processes were considered as significantly changed (FDR ≤ 0.05) according to the analysis of mitochondrial genes during the perinatal period—16th foetal day to the 4th postnatal day. No such biological process was identified in skeletal muscle.

GO Term	Genes	Enrichment	Count
Fatty acid β-oxidation using acyl-CoA dehydrogenase	*Acox2, Gcdh, Acadsb, Acadm, Acads, Acadl, Acox3, Acadvl, Ivd, Etfdh, Acad10, Etfb, Etfa*	9.68	13
Protein import into mitochondrial matrix	*Grpel1, Tomm7, Pam16, Timm17a, Tomm40l, Tomm20, Tomm40, Dnlz, Tomm22, Timm50, Timm44, Timm21*	6.54	12
Mitochondrial fission	*Mff, Fis1, Dnm1l, Opa1, Mief1, Mtfp1, Mul1, Park2, Ppp2r2b, Mtfr1l, Mtfr1*	6.17	11
Long-chain fatty acid metabolic process	*Slc27a1, Acsl1, Cd36, Acot2, Slc27a3, Acsl4, Acsl3, Slc27a2, Cpt1a, Acsl5*	3.9	10
ATP metabolic process	*Atp5d, Atp5e, Ndufaf7, Atp5b, Ak3, Ak2, Atp5g1, Ak4, Bad, Atp6v1a, Slc25a25, Atp5l, Atp5o, Atp5a1, Atp5i, Atp5h, Ndufs1*	3.88	17
NADH metabolic process	*Gpd2, Gpd1, Dlst, Idh3g, Idh3b, Ogdh, Idh3a, Mdh2, Mdh1*	3.53	9
Release of cytochrome c from mitochondria	*Mff, Bak1, Fis1, Dnm1l, Bcl2, Bax, Bcl2a1, Tp53, Mapk9, Timm50, Bad, Bcl2l1*	3.17	12
Tetrahydrofolate metabolic process	*Mthfd1, Shmt1, Mthfd2, Tyms, Mthfs, Shmt2, Mthfd1l*	3.08	7
Positive regulation of mitochondrial Ca^2+^ concentration	*Micu1, Fis1, Mcur1, Micu2, Rap1gds1, Tgm2, Mcu, Bcap31*	2.98	8
Glutathione metabolic process	*Gsta4, Aldh5a1, Ethe1, Clic1, Sod1, Hagh, Gsr, Gpx1, Clic4, Gstk1, Gpx4, Idh1, Txnrd1, Gstp1, Mgst1*	2.86	15

**Table 3:** Genes involved in CoQ biosynthesis. GO:0006744 (ubiquinone biosynthetic process) was not detected as significantly enriched among mitochondrial genes either in the rat liver or skeletal muscle (FDR > 0.05).

Human gene	Rat gene	Function
*PDSS1 * *	*Pdss1*	polyisoprenoid chain synthesis
*PDSS2 * *	*Pdss2*	polyisoprenoid chain synthesis
*COQ2 * *	*Coq2* ^†^	*p*-HB prenylation
*COQ3*	*Coq3* ^†^	modification step—O-methylation
*COQ4 * *	*Coq4* ^†^	scaffold protein
*COQ5 * *	*Coq5* ^†^	modification step—C-methylation
*COQ6 * *	*Coq6* ^†^	modification step—C5-hydroxylation
*COQ7 * *	*Coq7* ^†^	modification step—hydroxylation
*COQ8A * *	*Coq8a* ^†^	ATPase/kinase
*COQ8B * *	*Coq8b*	ATPase/kinase
*COQ9 * *	*Coq9* ^†^	lipid binding/scaffold protein/C4-hydroxylation
*COQ10A*	*Coq10a*	lipid or CoQ-intermediate binding
*COQ10B*	*Coq10b*	lipid or CoQ-intermediate binding
*NDUFA9*	*Ndufa9* ^†^	subunit A9 in NADH:ubiquinone oxidoreductase (complex I)
*UBIAD1*	*Ubiad1* ^†^	cholesterol and phospholipid metabolism

* Known primary CoQ_10_ deficiency in human; ^†^ Genes which are annotated in GO:0006744 (ubiquinone biosynthetic process) and were present on the array in this study.

## Data Availability

The microarray datasets generated and analysed during the current study are available in the NCBI’s Gene Expression Omnibus repository, (https://www.ncbi.nlm.nih.gov/geo/query/acc.cgi?acc=GSE131012 accessed date on 1 May 2021). Other data are available from the corresponding author on reasonable request.

## Supplementary Materials

The following are available online at https://www.mdpi.com/article/10.3390/biology10050418/s1, Table S1: Tables showing the significantly changed GO categories for every mentioned profile and/or the tables of gene symbols co-clustered in microarray GO and STEM analysis; Table S2: A table showing probes that were significantly changed in both the rat liver and skeletal muscle according to TIGR MeV, betr package analysis; Table S3: Table showing probes that were significantly changed in the rat liver or skeletal muscle only, according to TIGR MeV, betr package analysis; Table S4: Table of 248 unique genes that were significantly changed in both tissues regardless of tissue origin according to TIGR MeV, betr package analysis.

## Abbreviations

ATPase: ATP synthase; cDNA: Complementary DNA; CoQ: Coenzyme Q; Ct: Threshold cycle; ETC: Electron Transport Chain; F22: 22nd Foetal day; FDR: False Discovery Rate; *g*: centrifugal force (number of times the gravitational force); GEO: Gene Expression Omnibus; GO: Gene Ontology; miRNA: Small non-coding RNA molecule (microRNA); mRNA: Messenger RNA; mtDNA: Mitochondrial DNA; NADH: Nicotinamide Adenine Dinucleotide (reduced form); nDNA: Nuclear DNA; OXPHOS: Oxidative Phosphorylation System; P4: fourth Postnatal day; RIN number: RNA integrity number; STEM: Short Time-series Expression Miner.

## Appendix A

**Table A1:** STEM analysis of microarray data. STEM analysis defined 81 types of expression profiles. Each gene was aligned with certain expression profile. Clusters with more genes than expected were found to be significant (*p* ≤ 0.05). In the liver, there were 4 clusters with a significantly enriched number of genes identified (Clusters 0–3). In skeletal muscle, there were six clusters identified (Clusters 0–5). Genes which were clustered by STEM are available in the Supplementary data files [47].

Cluster Number	Liver	Skeletal Muscle
Cluster 0		
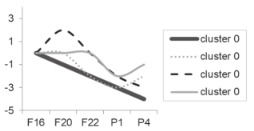
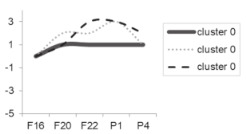
Cluster 1		
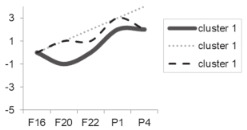
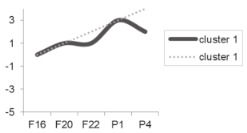
Cluster 2 Cluster 3		
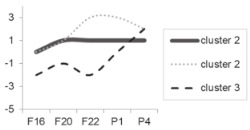
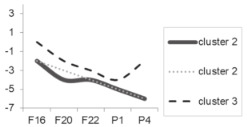
Cluster 4 Cluster 5	Not determined.	
